# Identification of YAP regulators through high-throughput screening and NanoBiT-based validation-drug repositioning for cancer therapy

**DOI:** 10.1080/19768354.2025.2489389

**Published:** 2025-05-08

**Authors:** Ji-Youn Lim, Eui-Hwan Choi, Yujeong Kim, Minseong Kim, Dongkyu Choi, Wantae Kim, Boksik Cha

**Affiliations:** a New Drug Development Center, Daegu-Gyeongbuk Medical Innovation Foundation, Daegu, Republic of Korea; b Department of Life Science, University of Seoul, Seoul, Republic of Korea; c Department of Biopharmaceutical Engineering, Hannam University, Daejeon, Republic of Korea; d KNU G-LAMP Project Group, KNU Institute of Basic Sciences, School of Life Sciences and Biotechnology, BK21 FOUR KNU Creative BioResearch Group, Kyungpook National University, Daegu, Republic of Korea; e Department of Biochemistry, Chungnam National University, Daejeon, Republic of Korea

**Keywords:** YAP, NanoBiT, thioridazine, vinorelbine, high-throughput screening

## Abstract

Yes-associated protein (YAP), a key co-transcription factor of the Hippo pathway, is a promising drug target for cancer therapy due to its critical role in promoting cell proliferation, survival, and tumor progression when dysregulated. While most Hippo pathway-targeting drugs focus on disrupting TEAD-YAP interactions or modulating the MST or LATS kinase cascade, new approaches are needed to identify small molecules that regulate YAP activity. In this study, we conducted high-throughput screening of FDA-approved drugs to discover potential YAP modulators. Using a NanoBiT-based system, which enables real-time and quantitative measurement of protein interactions, combined with phenotype-based assays in EGFP-YAP-expressing cells, we identified compounds that activate or inhibit YAP function. Among the identified YAP regulators, the microtubule destabilizer vinorelbine promoted YAP nuclear localization and transcriptional activation, while the antipsychotic drug thioridazine enhanced YAP phosphorylation at Ser127, resulting in its cytoplasmic retention and reduced transcriptional activity, effectively suppressing cancer cell growth. These findings demonstrate the potential of FDA-approved drugs in modulating YAP activity and present a novel screening strategy for developing YAP-targeting therapeutics. Furthermore, this approach can be extended to identify modulators of other signaling pathways, advancing drug discovery for a wide range of diseases.

## Introduction

The Hippo signaling pathway regulates organ size, cell proliferation, and tumor suppression by controlling a kinase cascade that phosphorylates YAP (Jang et al. 2023) Phosphorylated YAP is retained in the cytoplasm and degraded, preventing its transcriptional activation of genes involved in cell growth and survival. In contrast, when the Hippo pathway is inactive, unphosphorylated YAP translocates to the nucleus, where it interacts with TEAD transcription factors to drive gene expression and promote cell proliferation. (Pan 2007; Russell and Camargo 2022). YAP, along with its paralog TAZ, promotes the transcription of genes essential for cell survival and proliferation, such as connective tissue growth factor (CTGF), cysteine-rich angiogenic inducer 61 (CYR61), and ankyrin repeat domain 1 (ANKRD1), via interaction with TEAD transcription factors (Zhao et al. 2008; Pan 2010).

In canonical Hippo pathway, YAP are regulated by phosphorylation of the kinase cascade which composed by the serine/threonine protein kinases MST1 and MST2 (MST1/2) and the serine/threonine protein kinases LATS1 and LATS2 (LATS1/2). LATS1/2 directly phosphorylates YAP at S127, resulting in the cytoplasmic retention and degradation of YAP (Xie et al. 2022b). Dysregulation of YAP has been implicated in various cancers, including liver, breast, lung, and colorectal cancers, making it an attractive therapeutic target. However, the development of direct YAP inhibitors has proven challenging due to its complex regulation and lack of enzymatic activity (Zanconato et al. 2015; Calses et al. 2019; Luo et al. 2023). Drug repositioning – repurposing existing FDA-approved drugs – offers a practical strategy for identifying new therapeutic candidates that modulate YAP signaling, as it significantly reduces the time and cost associated with de novo drug discovery, while utilizing the established safety profiles of approved drugs (Kim et al. 2019; Kim et al. 2022).

Given the complexity of YAP regulation, traditional screening methods have faced limitations in efficiently identifying novel regulators. In this study, we conducted a high-throughput screen of FDA-approved drugs to identify compounds that regulate YAP activity. Using a combination of phenotype-based screening and NanoBiT technology, we discovered and validated potential YAP modulators, including vinorelbine and thioridazine. These findings provide new insights into YAP regulation and suggest novel therapeutic strategies for YAP-driven cancers.

## Materials and methods

### Generation of stable cell line

EGFP-YAP stable cell line was generated from HEK293A. HEK293A cells were seeded in 100 mm plates and transfected with EGFP-YAP and pBabe-puroR. Media was changed with DMEM in 4 h. After 24 h, media was replaced with DMEM containing puromycin (2.5μg/ml) for selection. Cells were grown for 7 days, replacing selective medium every 3 days. Colonies were isolated on 96 well with 200p tips that cut off the end. Stable cell lines are chosen according to the selection criterion.

### Dual luciferase assay

HEK293 cells were seeded in 96 well plates and transfected with Renilla and 8xGTIIC-Luc in each well. 4 h later, media was changed with fresh media containing drug. After 24 h, cells were lysed with Passive Lysis Buffer (Promaga) for 10 min at RT. Firefly and Renilla luciferase activity were measured by Synergy™ Neo (BioTek) using the dual-luciferase reporter assay kit (Promega) according to the manufacturer’s instructions. Firefly luciferase activity was normalized on Renilla luciferase activity.

### FDA approved drug library screening

A high-throughput screening approach was employed to identify compounds that modulate YAP localization using a library of 770 FDA-approved drugs (BML-2843, Enzo Biochem). EGFP-YAP-expressing stable cell lines were seeded in 96-well plates and treated with each drug at a concentration of 5μM for 24 h. The subcellular distribution of EGFP-YAP was analyzed using the Operetta CLS High-Content Analysis System, which allowed for automated fluorescence microscopy imaging and quantification. Image analysis was performed to distinguish between nuclear and cytoplasmic regions of each cell. The EGFP fluorescence intensity was measured in both compartments, and the nuclear-to-cytoplasmic ratio of EGFP-YAP was calculated to quantify YAP nuclear translocation. Compounds were classified based on their effect on YAP localization: drugs that increased the nuclear-to-cytoplasmic EGFP-YAP ratio to 0.54 or above were designated as YAP activators, while those that decreased the ratio to 0.51 or below were categorized as YAP inhibitors.

### Immunofluorescence

Cells were seeded onto PhenoPlate™ microplates and treated experimental conditions, gently washed three times with phosphate-buffered saline (PBS) to remove any residual media. Fixation was performed by incubating cells with 4% paraformaldehyde (PFA) in PBS for 15–20 min at room temperature. After fixation, the cells were washed again three times with PBS and subsequently permeabilized by incubation with 0.1% Triton X-100 in PBS for 10 min at room temperature. For blocking, cells were incubated in 5% bovine serum albumin (BSA) in PBS for 1 h at room temperature. After blocking, the cells were incubated overnight at 4°C with primary antibodies specific to the proteins of interest, diluted in the blocking solution according to the manufacturer’s recommendations. Following the primary antibody incubation, cells were washed three times with PBS to remove unbound antibodies. For secondary antibody labeling, cells were incubated with Alexa Fluor-conjugated secondary antibodies, diluted appropriately in the blocking solution, for 1 h at room temperature in the dark. Cells were washed three additional times with PBS to remove excess secondary antibodies. In the case of EGFP-YAP stable cell lines, primary and secondary antibody staining was not required, as the fluorescence signal was intrinsic to the EGFP tag. After the final wash, ProLong™ Diamond Antifade Mountant was used to preserve fluorescence for image analysis. Fluorescent images were captured and analyzed using the Operetta CLS High-Content Analysis System equipped with a confocal module. Appropriate excitation and emission filters were selected based on the fluorophores used in the experiment. High-resolution images were acquired with a 63x objective lens, and multiple fields per well were imaged to ensure representative sampling of the cell population. Quantitative analysis of fluorescence intensity and subcellular localization was performed using Harmony software, integrated with the Operetta CLS system.

### Western blot analysis

Proteins were isolated from HEK293 cells treated with indicated conditions. Cells were lysed with lysis buffer (containing 1% Triton X-100, 0.5% sodium deoxycholate, 0.1% SDS, and protease and phosphatase inhibitors) for 30 min. Lysate were collected after centrifugation at 12,000 × g for 10 min at 4°C and boiled with 4X loading dye at 95°C for 10 min for denaturation. Proteins were separated by SDS-PAGE electrophoresis and transferred to PVDF membranes. The membranes were incubated with primary antibodies and secondary HRP-conjugated antibodies. The protein bands were visualized with a chemiluminescence imaging system using chemiluminescence (ECL). Antibody for this study: Anti-phospho-YAP (Ser127) (#13008), YAP (#14074), GAPDH (#2118) were purchased from Cell Signaling Technology and β-actin (Sigma, A5441) was purchased from Sigma.

### Quantitative RT-PCR

Total RNA was extracted using the RNeasy mini kit (Qiagen, Cat#74104). Complementary DNA cDNA was synthesized from 2μg of total RNA with reverse transcriptase using TOPscriptTM RT DryMIX (Enzynomics) according to the manufacturer’s protocol. For quantitative real-time PCR, each cDNA was quantified by QuantStudio1 (Applied biosystem) using 2xReal-Time PCR Master Mix including SFCgreen (Biofact) and normalized to GAPDH/β-ACTIN.

The primer sequences used for RT-qPCR

hGAPDH For: 5’-AGCCACATCGCTCAGACAC-3’

hGAPDH Rev: 5’-GCCCAATACGACCAAATCC-3’

hβ-ACTIN For: 5’-TCACCCACACTGTGCCCATCTACGA-3’

hβ-ACTIN Rev: 5’-CAGCGGAACCGCTCATTGCCAATGG-3’

hCTGF For: 5’-AGGAGTGGGTGTGTGACGA-3’

hCTGF Rev: 5’-CCAGGCAGTTGGCTCTAATC-3’

hCYR61 For: 5’-CCTTGTGGACAGCCAGTGTA-3’

hCYR61 Rev: 5’-ACTTGGGCCGGTATTTCTTC-3’

hANKRD1 For: 5’-AGTAGAGGAACTGGTCACTG-3’

hANKRD1 Rev: 5’-TGGGCTAGAAGTGTCTTCAG-3’

### Nanobit cell-based immunoassay

For NanoBiT assay, cells were cultured a concentration of 5 × 10^4^ cells/ml per 96-well plates in 150μl of complete growth medium for overnight. The medium was replaced with serum-free medium once cells adhered stably and incubated overnight. For treatments, the medium was replaced with 30μl containing inhibitors. To lyse cells, 10μl of lysis buffer (0.1% digitonin in 1× immunoassay buffer with protease and phosphatase inhibitors) was added, and plates were mixed for 30 min. Lysates were then combined with 50 µl of antibody mix (0.3 μl of each primary and NanoBiT detection antibody per well, diluted in 1× immunoassay buffer) and incubated at roomtemperature for 2hr. Subsequently, 25μl of Nano-Glo reagent (Nano-Glo Luciferase Assay Substrate in 1× immunoassay buffer) was added, and luminescence was recorded using a plate luminometer.

### Synergism of cytotoxic effects

The synergistic cytotoxic effects of thioridazine and vinorelbine were evaluated using HeLa and HCT116 cell lines. Cells were seeded at a density of 2,000 cells per well in 96-well plates and allowed to stabilize for 24 h at 37°C in a humidified incubator with 5% CO₂. Following stabilization, cells were treated with a combination of thioridazine (0–10μM) and vinorelbine (0–5 μM) for 72 h. Cell viability post-treatment was measured using a cck-8, according to the manufacturer's instructions. Absorbance values were used to compute the fraction of cells affected (Fa) and fraction of cells unaffected (1-Fa), which were input into the CalcuSyn software for CI analysis. The combination effects of the two drugs were assessed using the CalcuSyn software (BIOSOFT), which performs dose-effect analysis based on the combination index (CI) method as described by Chou and Talalay. This method quantifies drug interactions as synergistic (CI < 1), additive (CI = 1), or antagonistic (CI > 1). Dose–response curves for each drug alone and in combination were generated, and CI values were calculated at GI_50_ to evaluate synergism at this specific growth inhibition level.

## Result

### Identification of YAP modulators through FDA-Drug library screening in EGFP-YAP stable cells

To identify small molecules that regulate YAP, we generated a stable HEK293A cell line that stably expresses EGFP-YAP, which allows for the visualization of YAP localization. The following experiments were conducted to verify the suitability of the EGFP-YAP stable cell line for screening novel regulators of YAP. Initially, cells were seeded into a 96-well plate. As shown in Supplementary Figure 1A, increasing cell density resulted in a significant decrease in YAP's nuclear localization, accompanied by an increase in its cytosolic intensity (Figure S1B). This shift in localization was corroborated by an increase in YAP phosphorylation (Figure S1C) and a corresponding decrease in the expression of well-established downstream targets genes such as CTGF and CYR61 (Figure S1D). Their expression levels are directly influenced by YAP’s transcriptional activity, making them reliable indicators of YAP’s functional status within cells (Reddy et al. 2013). Cell density significantly influences YAP localization and activity, as it induces cytoskeletal tension changes that regulate YAP’s nuclear-cytoplasmic distribution (Piccolo et al. 2023). As shown in Supplementary Figure 1E, treatment with Dasatinib, a well-characterized YAP-modulating compound, resulted in a clear shift in YAP localization, confirming the reliability of our system for detecting YAP modulators. The quantitative data further support these observations, showing a significant change in the nuclear-to-cytoplasmic ratio of YAP. These findings validate the suitability of the established EGFP-YAP stable cell system for accurately measuring YAP activity.

**Figure 1.**
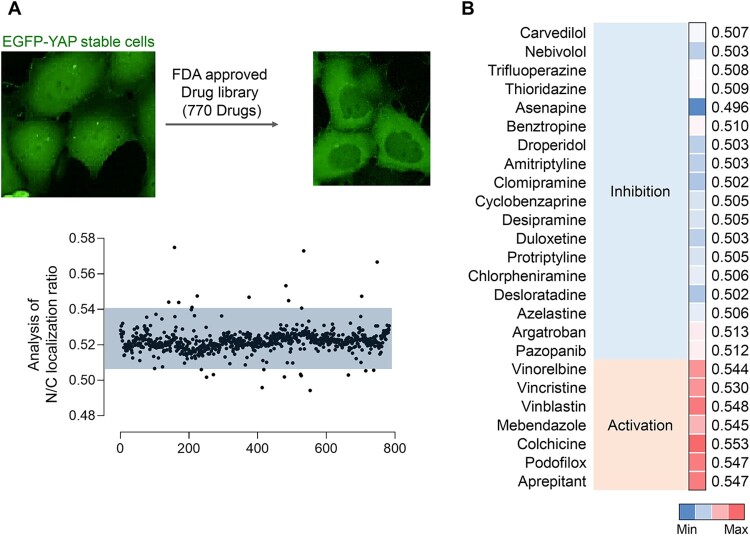
High-throughput screening for the YAP regulator. (A) Scheme of high-throughput screening to analyze YAP localization on EGFP-YAP stable cell line. Analysis of the YAP nuclear/cytoplasm ratio was implemented in graph for 770 FDA approved drugs. (B) Heatmap of drugs that upregulate or downregulate YAP/TAZ in high-throughput screening.

Then, we proceeded to evaluate a library of 770 FDA-approved drugs using EGFP-YAP stable cell line to identify potential YAP-regulating compounds. The high-throughput screening was designed to analyze the overall distribution and identify drugs that affect the positional dynamics of YAP between the nucleus and cytoplasm (Figure 1A). This screening approach allowed us to evaluate compounds for their ability to modulate YAP localization and provided insights into their potential mechanisms of action. Notably, microtubule inhibitors such as vinorelbine, vincristine, vinblastine, mebendazole, colchicine, and podofilox were found to promote YAP activation (Figure 1B).

### Microtubule inhibitors as a YAP regulator

Microtubule-targeting agents (MTAs), which influence microtubule dynamics, are broadly classified into two categories: microtubule-stabilizing agents (MSAs) and microtubule-destabilizing agents (MDAs) (Borys et al. 2020). MSAs, such as paclitaxel, promote microtubule polymerization, whereas MDAs, like vinca alkaloids, disrupt microtubule formation by depolymerization (Jordan and Wilson 2004). Given that microtubule dynamics play a critical role in maintaining cytoskeletal integrity, these agents may indirectly influence YAP activity by altering cytoskeletal tension, a key regulator of YAP localization and function. We investigated the effects of microtubule destabilizers and stabilizers on YAP localization and activity using EGFP-YAP stable cells under varying cell density conditions.

**Figure 2.**
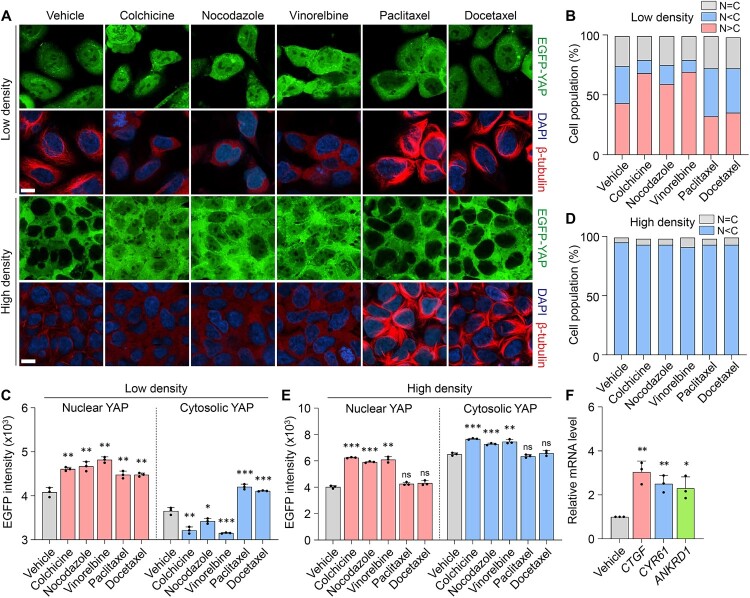
Microtubule inhibitors increase YAP and TAZ activities through blocking degradation. (A) Representative confocal images of 5μM drug in EGFP-YAP stable cells at low or high density. (Scale bar: 10μm) (B) The ratio of EGFP-YAP refers to the relative degree of fluorescence. The percentage of the cell population was counted according to the specified ratio at low density. (C) Data represent the nuclear and cytosolic YAP intensity within cell compartments in each drug at low density (6 × 103 cells/well). (D) The experiment for percentage of the cell population was also conducted at high density (5 × 104 cells/well). (E) Data represent the fluorescence intensity of nuclear and cytosolic YAP within cell compartments in each drug at high density (5 × 104 cells/well). (F) HEK293 were treated with 5μM vinorelbine. mRNA levels measured with RT-qPCR and normalized to GAPDH mRNA levels. *P*-values determined by unpaired t-tests for multiple testing. (*P < 0.05, **P < 0.01, and ***P < 0.001.)

Our findings show that microtubule destabilizers promote YAP nuclear localization and activity, whereas stabilizers exhibit more complex regulatory patterns, suggesting distinct mechanisms of action. EGFP-YAP stable cells were treated under low and high cell density conditions using three microtubule destabilizers (colchicine, nocodazole, and vinorelbine) and two stabilizers (paclitaxel and docetaxel) (Rhee et al. 1997). The effects on YAP localization were visualized through immunofluorescence microscopy with EGFP-YAP shown in green, nuclei in blue (DAPI), and ß-tubulin in red (Figure 2A). In low-density conditions, microtubule destabilizers significantly increased the nuclear localization of YAP, as indicated by the enhanced intensity of nuclear YAP (Figure 2A, top row). Quantitative analysis demonstrated that the proportion of cells exhibiting nuclear YAP localization was significantly elevated following treatment with destabilizers in low-density conditions (Figure 2B). Specifically, nuclear YAP intensity was significantly elevated, whereas cytosolic YAP levels were relatively reduced (Figure 2C). In contrast, stabilizer treatment under the same conditions led to increased total YAP levels, reflected in both nuclear and cytosolic compartments (Figure 2A-C). Under high-density conditions, where YAP is typically sequestered in the cytoplasm, microtubule destabilizer treatment still enhanced YAP intensity in both the nucleus and cytoplasm (Figure 2A, bottom row). This was confirmed by quantifying the proportion of cells with predominant nuclear YAP (Figure 2D) and by measuring nuclear and cytosolic YAP intensities (Figure 2E). Interestingly, stabilizers did not significantly affect YAP translocation in high-density conditions, maintaining the cytosolic retention of YAP (Figure 2A, D, E). These data suggest that even the same microtubule inhibitor can have different regulatory effects depending on its mechanism of action.

To confirm the activating effect of microtubule stabilizers and destabilizers on endogenous YAP, we treated the microtubule inhibitors on HCT116 and immunostained the YAP and its paralog TAZ (Figure S2A). Consistent with the previous experiment, there is a change in YAP/TAZ activation. Regardless of cell density, microtubule destabilizers promoted the nuclear localization of YAP/TAZ (Figure S2A-E). Moreover, YAP activation by microtubule stabilizers was attenuated in low-density conditions (Figure S2B-C). These results demonstrate the complex effects of microtubule dynamics on YAP activity. Microtubule destabilizers consistently enhance YAP nuclear localization and activation, indicating that microtubule destabilization triggers YAP activation through cytoskeletal remodeling (Zhao et al. 2012). In contrast, the impact of microtubule stabilizer varies depending on conditions such as cell density. Under low-density conditions, microtubule stabilizers tend to attenuate YAP activation. This suggests that microtubules stabilization may stimulate the Hippo pathways to inhibit YAP under specific condition (Li et al. 2023). To further validate the observations, we extended our analysis to various cell lines, including 293A, HeLa, sh-sy-5y, and PC9. We assessed relative mRNA levels of YAP target genes (CTGF, CYR61, and ANKRD1) after vinorelbine treatment (Xie et al. 2022b). Consistent with our primary findings, vinorelbine elevated target gene expression across all cell lines (Figure 2F, S2F), supporting the hypothesis that microtubule destabilizers robustly activate YAP signaling.

### Identification of novel FDA-approved drugs that negatively regulate YAP localization and target gene expression

Following the high-throughput screening detailed in Figure 1, where several FDA-approved drugs were identified as potential YAP inhibitors, further experiments were conducted to confirm and characterize the inhibitory effects of these compounds. In the initial high-throughput screening, 14 FDA-approved drugs were identified as potential YAP inhibitors, spanning several drug classes: Beta-Blockers (Carvedilol, Nebivolol), Antipsychotics (Thioridazine, Asenapine, Droperidol), Antidepressants (Amitriptyline, Clomipramine, Cyclobenzaprine, Desipramine, Duloxetine, Protriptyline), and Antihistamines (Chlorpheniramine, Desloratadine, Azelastine).

**Figure 3.**
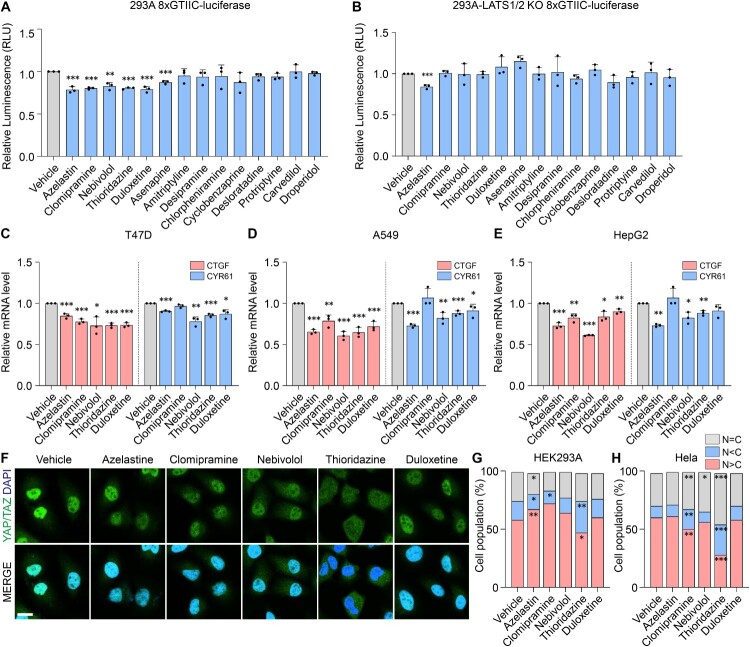
YAP is regulated Hippo-LATS dependently by amino acid transporters. (A) HEK293A cells were transfected with RL-null and 8xGTIIC-luciferase treated with 5μM of 14 FDA-approved drugs. (B) HEK293A LATS1/2 knockout cells were transfected with RL-null and 8xGTIIC-luciferase. Luciferase activity was measured with the indicated drug for 5μM. (C-E) Relative transcriptional activity is analyzed by RT-qPCR in (C) T47D, (D) A549, and (E) HepG2. (F) Confocal images of immunofluorescence YAP/TAZ in Hela cells 5μM indicated drugs for 24 hr. (Scale bar: 10μm) (G, H) The ratio of cell population refers to represent the relative intensity of YAP/TAZ localization. The percentage of the cell population was counted according to the specified criteria. Each of indicated drugs was administered on (G) HEK293A, (H) Hela. *P*-values determined by unpaired t-tests for multiple testing. Quantification data represent the mean ± SD of three independent experiments. (*P < 0.05, **P < 0.01, and ***P < 0.001.)

To confirm the regulatory effects of these compounds on YAP activity, we performed a luciferase reporter assay using the 8xGTIIC-luciferase construct, which is responsive to YAP/TAZ transcriptional activity. Treatment with YAP-inhibitory drugs resulted in a significant reduction in luciferase activity, indicating suppressed YAP transcriptional function. Among the tested drugs, Azelastine, Chlorpheniramine, Nebivolol, Thioridazine, and Duloxetine showed strong inhibitory effects on YAP activity (Figure 3A). To determine whether this inhibition was dependent on upstream Hippo signaling, the assay was repeated in LATS1/2 knockout 293A cells. In the absence of LATS1/2, the inhibitory effects of most compounds were attenuated, suggesting that their impact on YAP activity is largely dependent on upstream kinase signaling (Figure 3B). Notably, Azelastine maintained a strong inhibitory effect even in LATS1/2 knockout cells, indicating that it can suppress YAP activity independently of LATS1/2 phosphorylation. Next, we performed real-time PCR to measure the expression levels of YAP target genes CTGF and CYR61 in multiple cancer cell lines, including T47D, A549, and HepG2, following treatment with the five selected drugs (Figure 3C-E). The results showed a significant reduction in CTGF and CYR61 mRNA levels, particularly in response to Azelastine, Nebivolol, and Thioridazine across all three cell lines. To further investigate the effects on YAP/TAZ localization, immunofluorescence microscopy was conducted. Thioridazine, in particular, significantly promoted the cytoplasmic retention of YAP/TAZ, reducing their nuclear localization. This effect was consistently observed in the 293A cell line as well (Figure 3F-G). We further confirmed that Thioridazine effectively inhibits endogenous YAP translocation in HeLa (Figure 3H).

### Nanobit cell-based immunoassay for measuring YAP activity

**Figure 4.**
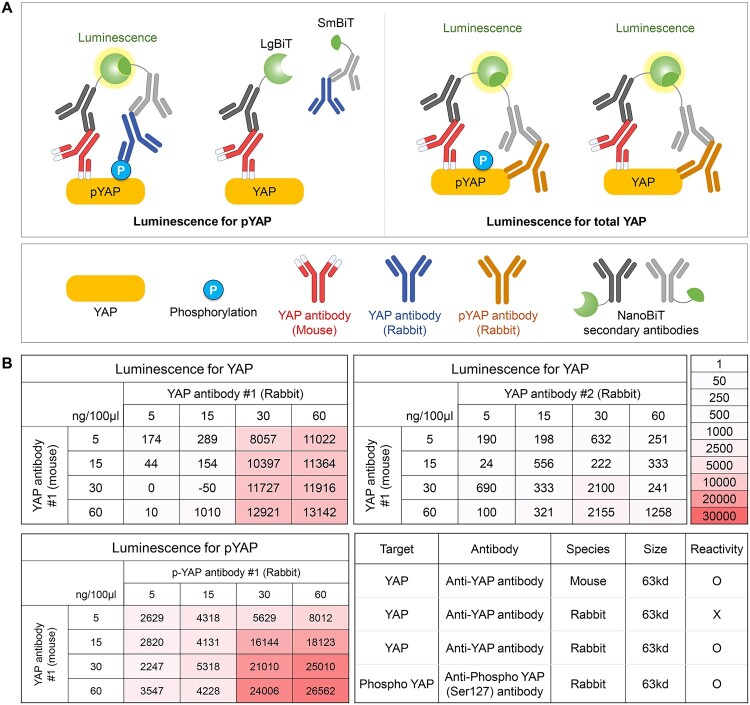
Development of the NanoBiT based immuno-screening (A) A brief scheme for NanoBiT based immunoassay. (B) For the immunofluorescence analysis, the level of phosphorylated YAP was measured by NanoBiT-based immunoassay using the indicated antibody pairs at the indicated concentrations after addition of antibodies. A heat map represents the calculated fold change.

Recently, NanoBiT-based bioluminescence technology has emerged as a powerful tool for studying protein–protein interactions with high sensitivity (Dixon et al. 2016). This method relies on the complementation of two non-active luciferase subunits, LargeBiT (LgBiT; 18 kDa) and SmallBiT (SmBiT; 11 amino acid peptide), which emit light only when brought into close proximity by interacting proteins (Hwang et al. 2020; Liu and Guo 2022) (Figure 4A). Utilizing this principle, we applied the NanoBiT system to develop a sensitive assay for measuring YAP activity by tagging phospho-specific and general YAP antibodies with LgBiT and SmBiT, respectively. In this approach, a phospho-specific antibody that recognizes YAP phosphorylation at Ser127 is conjugated to one NanoBiT subunit (SmBiT), while a general YAP antibody, which binds total YAP, is tagged with the complementary subunit (LgBiT). When phosphorylated YAP is present, the two antibodies – one specific to phosphorylated YAP and the other recognizing total YAP – bind to the same YAP molecule, bringing LgBiT and SmBiT into close proximity and generating a bioluminescent signal. This system allows for real-time, quantitative detection of phosphorylated YAP with exceptional specificity and sensitivity (Hwang et al. 2023). The success of such an immunoassay critically depends on the quality and specificity of the primary antibodies used, as these determine the accuracy and reliability of phospho-YAP detection (Xie et al. 2022a).

To demonstrate the feasibility of the NanoBiT cell-based immunoassay for detecting YAP phosphorylation, we optimized an antibody pair for use in a luminescence-based detection system. The assay design incorporates a phospho-specific antibody that selectively binds phosphorylated YAP at Ser127, enabling luminescence to quantitatively reflect phosphorylation levels (Figure 4A). Phosphorylation of YAP at Ser127 (or TAZ at Ser89) facilitates its interaction with 14-3-3 proteins, leading to cytoplasmic retention and inhibition of transcriptional activity, which is a key regulatory mechanism in the Hippo pathway (Liu et al. 2010; Zhao et al. 2010; Huang et al. 2012; Kim and Jho 2018). The optimization process involved screening commercially available mouse and rabbit antibody pairs. A phospho-YAP (Ser127) antibody was paired with a secondary antibody recognizing a different YAP epitope. Each combination was titrated across a concentration matrix and evaluated for its ability to detect phospho-YAP and total YAP in HEK293 cells. Among the tested pairs, the combination of p-YAP rabbit antibody #1 and YAP mouse antibody #1 yielded the highest luminescence signal for phospho-YAP, while the YAP rabbit antibody #1 and YAP mouse antibody #1 pair was optimal for detecting total YAP (Figure 4B). In contrast, the YAP rabbit antibody #2 paired with YAP mouse antibody #1 produced lower luminescence, emphasizing the critical importance of antibody selection. Based on these results, we selected the optimal antibody pair (#1) at a concentration of 300 ng/mL for further experiments. This optimized NanoBiT cell-based assay demonstrated high sensitivity and specificity for detecting both phosphorylated and total YAP, providing a robust system for monitoring YAP activity under various experimental conditions.

### Validation of NanoBiT immunoassay sensitivity for quantifying phosphorylated and total YAP

**Figure 5.**
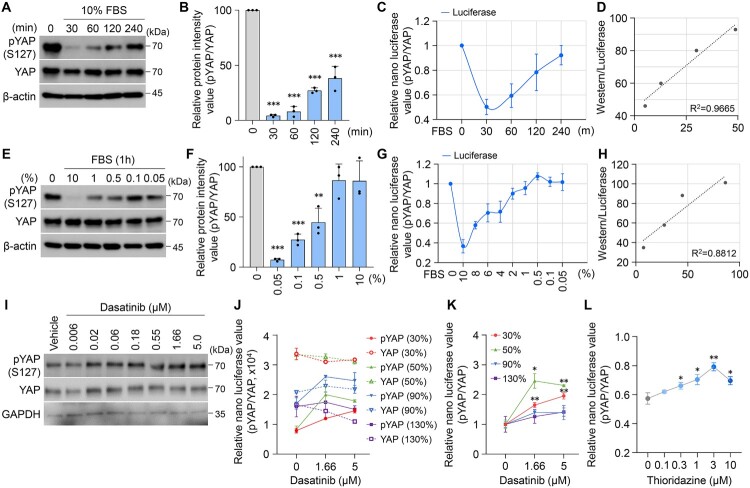
Serum induces YAP dephosphorylation (A, E) HEK293 cells were starved in medium without fetal bovine serum (FBS) for 16hr and then stimulated with 10% FBS for the 30, 60, 120, and 240 min (A) or with different concentrations of FBS (0, 0.05, 0.1, 0.5, 1, and 10%) for 1hr (E), respectively. Cell lysates were subjected to western-blotting with the indicated antibodies. Where indicated, gels containing phosphortag were employed for assessment of YAP phosphorylation status. (B, F) The expression level of phosphorylated YAP was quantified by immunoblotting assay, with β-actin used as a reference control for normalization. (C, G) The levels of NanoBiT luciferase (C) for various time points and (G) for various concentration of FBS. Phosphorylated or total YAP levels were measured by the corresponding NanoBiT cellbased immunoassay. (D, H) Changes in gene expression analyzed by western-blot compared with luciferase assay results. The protein expression values of western-blot compared with normalized values in signal levels detected by luciferase analysis. (I) HEK293 cells were treated with diverse concentrations of Dasatinib. (J) The level of NanoBiT luciferase for different cell density with various concentration of Dasatinib. (K) Changes in expression levels of phosphorylated YAP were analyzed after diverse concentrations of Dasatinib treatment by NanoBiT luciferase with the total levels of YAP as a reference control for normalization. (L) Luciferase activity reflecting YAP phosphorylation was measured using the NanoBiT assay after Thioridazine treatment and normalized to total YAP at 50% confluency. The error bars are the mean ± SD from two independent biological replicates. a.u, arbitrary unit. Intensities were quantified by NIS advanced software (Nikon). (*P < 0.05, **P < 0.01, and ***P < 0.001.)

To validate the sensitivity of the NanoBiT immunoassay for detecting YAP activity, we measured FBS (fetal bovine serum)-dependent changes in YAP phosphorylation. Starvation and FBS treatment can have significant effects on YAP activity, as serum starvation increases YAP phosphorylation, promoting its cytosolic localization, whereas FBS stimulation reduces phosphorylation, leading to nuclear retention and transcriptional activation. In our experiments, the addition of serum led to a rapid and significant decrease in YAP phosphorylation by over 90% within 30 min, as demonstrated by immunoblotting with a phospho-specific YAP (Ser127) antibody (Figure 5A and B). Dose-dependent FBS stimulation (0%, 0.05%, 0.1%, 0.5%, 1%, and 10%) further confirmed a decrease in pYAP levels (Figure 5E and F). To corroborate these findings, we utilized the NanoBiT immunoassay, which showed results consistent with Western blotting. Serum starvation promoted YAP phosphorylation, while increasing serum concentrations restored YAP dephosphorylation over time (Figure 5C, G). Importantly, a strong positive correlation was observed between soluble pYAP protein levels and luminescence signals from the NanoBiT assay (Figure 5D, H), confirming the assay's accuracy and reliability.

It is well established that increased cell density activates the Hippo kinase pathway, leading to YAP phosphorylation, cytoplasmic retention, and degradation in vitro and in vivo. Since cell density uniquely regulates YAP activation, we further evaluated the NanoBiT assay’s ability to measure cell density-dependent changes in YAP activity, particularly in response to drug treatment. Dasatinib, a known YAP inhibitor that promotes YAP phosphorylation (Oku et al. 2015; Sun et al. 2018; Lamar et al. 2019) was used as a test compound. Western blot analysis revealed a dose-dependent increase in YAP phosphorylation following Dasatinib treatment for 6 h (Figure 5I). We then examined the effects of cell density on Dasatinib-mediated YAP regulation. HEK293 cells seeded at varying densities (30% to 130%) were treated with Dasatinib, and YAP phosphorylation levels were monitored using the NanoBiT assay. It was observed that the level of total YAP decreased as the density increased (Figure 5J dash line), while conversely, the level of phospho YAP increased (Figure 5J line). At low cell densities (30% and 50%), Dasatinib significantly increased phospho-YAP levels, which corresponded with a decrease in YAP transcriptional activity (Figure 5J, K). In contrast, at confluency levels exceeding 100%, the effect of Dasatinib on YAP phosphorylation was diminished (Figure 5J and K). This suggests that cell density attenuates Dasatinib’s inhibitory effect, likely due to increased cell–cell contact signaling that promotes baseline YAP inactivation in high-confluency cultures. Collectively, these findings demonstrate the robustness of the NanoBiT-based luciferase assay in evaluating dynamic changes in YAP phosphorylation and activity under different cellular conditions. The assay’s sensitivity to serum concentration, cell density, and drug responses highlights its potential for accurately assessing the efficacy of YAP-targeting compounds in diverse experimental contexts.

Finally, we evaluated the effects of Thioridazine on YAP phosphorylation using the NanoBiT assay. Treatment with Thioridazine significantly increased the ratio of phosphorylated YAP to total YAP (pYAP/YAP), indicating enhanced YAP phosphorylation and cytoplasmic retention, which corresponds to reduced YAP transcriptional activity (Figure 5L). These results are consistent with our previous findings that Thioridazine effectively suppresses YAP translocation and activity.

### Synergistic inhibition of cancer cell growth via YAP modulation by thioridazine and vinorelbine combination

Vinorelbine, while effective in disrupting microtubule dynamics and inducing cancer cell death, also activates YAP signaling, a known pro-survival mechanism that can counteract its anticancer efficacy. Our high-throughput screen revealed that multiple microtubule inhibitors, including vinorelbine, consistently promote YAP nuclear localization and transcriptional activation. To overcome this limitation, we selected thioridazine, an FDA-approved antipsychotic drug identified in our screen as a potent YAP inhibitor. Thioridazine enhances YAP phosphorylation at Ser127, promoting cytoplasmic retention and suppressing downstream gene expression. We hypothesized that thioridazine could counteract vinorelbine-induced YAP activation, thereby potentiating its anticancer activity.

**Figure 6.**
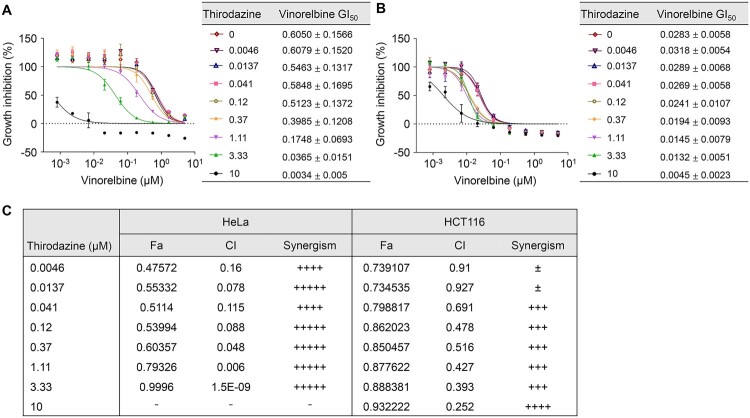
Synergism of cytotoxic effects of Vinorelbine and Thioridazine in HeLa and HCT116 cells. (A) Growth inhibition curve and GI50 values of Vinorelbine against Thioridazine at concentrations of 0-10uM in HeLa cells. Co-treatment with Thioridazine significantly reduced the Vinorelbine GI50 value, indicating enhanced cytotoxicity compared to Vinorelbine alone. (B) Growth inhibition curve and GI50 values of vinorelbine against thioridazine at concentrations of 0-10uM in HCT116 cells. Combined treatment showed greater growth inhibition, with reduced vinorelbine GI50 values compared to the single treatment. (C) Growth inhibition (Fa) values and synergistic effect analysis (CI) of Vinorelbine and Thioridazine in HeLa cells and HCT cells. Combination index of Thioridazine and Vinorelbine treatment was analyzed using CompuSyn software. The Fa values of HeLa cells inhibited by various combinations of Thioridazine concentrations at Vinorelbine 0.555uM ranged from 0.47572 to 0.99996, confirming cell growth inhibition, and the synergistic effect of the two drugs was confirmed by the CI values. For HCT116 cells, the Fa values of HeLa cells inhibited by various combinations of Thioridazine concentrations at Vinorelbine 0.0617uM ranged from 0.7391 to 0.9322 and the CI values indicate synergistic effects. Synergism levels were categorized based on the combination index (CI) values as follows: CI < 0.1 indicates +++++ (very strong synergism), 0.1–0.3 indicates ++++ (strong synergism), 0.3–0.7 indicates +++ (synergism), 0.7–0.85 indicates ++ (moderate synergism), 0.85–0.9 indicates + (slight synergism), and 0.9–1.1 indicates ± (nearly additive). These classifications provide a quantitative measure of the interaction between Thioridazine and Vinorelbin.

Given that Thioridazine and Vinorelbine exert opposing effects on YAP signaling – Thioridazine promotes YAP phosphorylation and cytoplasmic retention, whereas Vinorelbine enhances YAP nuclear localization and activation – we hypothesized that their combination could produce a synergistic effect to suppress cancer cell growth. To test this hypothesis, we evaluated the combined effect of these two drugs on YAP modulation and cancer cell growth in HCT116 and HeLa cell lines. When thioridazine was co-treatment at various concentrations (0.0046 μM - 10 μM), both cell lines exhibited a concentration-dependent significant decrease in the GI_50_ values of Vinorelbine. Notably, in HeLa cells, the GI_50_ of Vinorelbine decreased substantially from 0.6050 μM to 0.0034 μM (99.44% reduction) when treated with 10 μM thioridazine (Figure 6A). Similarly, in HCT116 cells, the GI50 decreased from 0.028 μM to 0.0045 μM (83.93% reduction) (Figure 6B), confirming synergistic effects in both cell lines and demonstrating varied responsiveness among different cell lines. To quantitatively assess the synergistic effect, we performed Combination Index (CI) analysis (Figure 6C). CI values less than 1 indicate stronger synergistic effects. Combination Index (CI) analysis is extensively discussed in pharmacological studies focusing on drug interactions. A CI value of less than 1 indicates synergy, meaning the combined effect of two drugs is greater than their individual effects combined (Roell et al. 2017; Repash et al. 2021). We utilized the Chou-Talalay model to analyze drug combinations, enabling us to identify synergistic, additive, or antagonistic interactions (Chou and Talalay 1984). In HeLa cells, most concentrations demonstrated very strong synergy (CI < 0.1) In HCT116 cells, CI values decreased as concentration increased, with the strongest synergy observed at 10 μM. We analyzed the Fraction affected (Fa) to assess the growth inhibition rate, evaluating the impact of the thioridazine and Vinorelbine combination on cell growth. The Fraction affected (Fa) metric provides a quantitative measure of cell growth inhibition, enabling a deeper understanding of the efficacy of drug combinations (Borys et al. 2020). The nearly complete inhibition observed suggests that thioridazine may enhance the efficacy of Vinorelbine by overcoming compensatory mechanisms often activated in cancer cells to evade apoptosis. In HeLa cells, Fa values increased with increasing thioridazine concentration, reaching a maximum of 0.9996 (99.96% growth inhibition) at 3.33 μM. This indicates that the drug combination can almost completely inhibit HeLa cell growth at this concentration. In addition, the HCT116 cell line also had an increased Fa value, confirming that cell growth was effectively inhibited in both cell lines, although reactivity was different. This indicates that the drug combination can almost completely inhibit HeLa cell growth at this concentration. These results collectively indicate that the combination of thioridazine and Vinorelbine exhibits a potent synergistic effect in inhibiting cancer cell growth by effectively modulating YAP signaling. This finding presents a promising new strategy for cancer treatment.

## Discussion

The findings of this study underscore the pivotal role of YAP modulation in cancer biology and highlight promising therapeutic opportunities (Reddy et al. 2013). By employing a high-throughput screening approach using an FDA-approved drug library, we identified several compounds, including microtubule inhibitors, that regulate YAP activity. This initial screening provided a broad overview of how various FDA-approved drugs influence YAP localization and activity, offering a strong foundation for further investigation into their mechanisms of action and therapeutic potential in YAP-driven cancers.

The NanoBiT-based approach further enhanced this study by facilitating real-time monitoring of dynamic changes in YAP localization and phosphorylation under various conditions. Unlike conventional static methods such as Western blotting, the NanoBiT system enables high-sensitivity, quantitative detection of YAP phosphorylation at Ser127, a key regulatory event leading to YAP cytoplasmic retention and inactivation (Xie et al. 2022). This system also proved scalable and well-suited for high-throughput applications, allowing rapid identification of potential YAP regulators and providing precise insights into YAP activity in response to drug treatments, mechanical cues, or cell density changes.

A notable discovery of this study is the synergistic effect between thioridazine and vinorelbine on cancer cell growth inhibition. Given that these two drugs exhibit contrasting effects on YAP signaling – vinorelbine promoting YAP nuclear localization and transcriptional activation, while thioridazine enhances YAP phosphorylation and cytoplasmic retention – we hypothesized that their combination might effectively suppress YAP-driven cancer growth. This hypothesis was supported by experimental results showing a significant reduction in the GI_50_ values of vinorelbine in both HeLa and HCT116 cell lines when combined with thioridazine. The strongest synergy was observed in HeLa cells, where vinorelbine’s GI_50_ was reduced by 99.44% when combined with thioridazine at 10μM (Figure 6A-B).

The contrasting mechanisms of these compounds provide complementary therapeutic opportunities. First, the complementary mechanisms of vinorelbine and thioridazine enable versatile therapeutic strategies for targeting various cancer subtypes or stages (Nair et al. 2023; Zhang et al. 2023). Vinorelbine, a microtubule destabilizer, promotes YAP nuclear localization and transcriptional activation, whereas thioridazine, a dopamine receptor antagonist, enhances YAP phosphorylation, leading to its cytoplasmic retention and transcriptional inhibition. These distinct mechanisms allow for targeting both YAP hyperactivation in aggressive cancers and contexts where YAP activation can induce apoptosis or differentiation, thus broadening therapeutic applicability across cancer types and stages (Cunningham and Hansen 2022; Zhao et al. 2023). Second, their synergistic effects reduce the likelihood of drug resistance by attacking the cancer cells through multiple pathways (Tsvetkova and Ivanova 2022; Meric-Bernstam et al. 2023). The synergistic interaction between vinorelbine and thioridazine simultaneously disrupts microtubule dynamics and inhibits YAP-mediated transcription. This dual approach prevents cancer cells from activating compensatory survival mechanisms that often lead to resistance against single-agent therapies, as shown in studies of combination treatments targeting complementary pathways (Jensen et al. 2020; Li et al. 2021; Batalha et al. 2023). Third, the differential effects of vinorelbine and thioridazine on YAP activity under varying cell density conditions suggest that therapeutic strategies can be tailored to tumor microenvironments with distinct cellular compositions (Choi et al. 2024). This context-dependent modulation of YAP activity may also influence chemosensitivity, as cell density can alter the cellular response to drugs by affecting signal transduction pathways and the microenvironment (Punyamurtula et al. 2023). For example, vinorelbine’s ability to enhance YAP activity in low-density regions and thioridazine’s inhibitory effect in high-density regions can be leveraged to optimize drug efficacy based on tumor characteristics.

The synergistic interaction between thioridazine and vinorelbine provides complementary therapeutic opportunities:

1. Distinct Mechanisms of Action: Vinorelbine, a microtubule destabilizer, induces YAP nuclear localization and activates YAP-dependent target genes, such as CTGF and CYR61, under low cell-density conditions (Reddy et al. 2013; Su et al. 2023). In contrast, thioridazine, a dopamine receptor antagonist, inhibits YAP transcriptional activity by promoting its phosphorylation at Ser127, leading to cytoplasmic retention (Zhou et al. 2023). These contrasting mechanisms enable versatile strategies to target both YAP hyperactivation in aggressive cancers and contexts where YAP activation induces apoptosis or differentiation (Cunningham and Hansen 2022; Zhao et al. 2023).
2. Reduced Drug Resistance: The combination of thioridazine and vinorelbine simultaneously disrupts microtubule dynamics and suppresses YAP-driven transcription, reducing the likelihood of drug resistance by targeting cancer cells through multiple pathways (Tsvetkova and Ivanova 2022; Meric-Bernstam et al. 2023). This approach prevents the activation of compensatory survival mechanisms often responsible for resistance in single-agent therapies (Jensen et al. 2020; Li et al. 2021; Batalha et al. 2023).
3. Context-Dependent Modulation: The differential effects of vinorelbine and thioridazine on YAP activity under varying cell density conditions suggest that this combination can be tailored to tumor microenvironments with distinct cellular compositions (Choi et al. 2024). Vinorelbine’s ability to enhance YAP activity in low-density regions and thioridazine’s inhibitory effect in high-density regions can be leveraged to optimize drug efficacy based on tumor characteristics (Punyamurtula et al. 2023).

In conclusion, our study provides a robust framework for understanding and manipulating YAP activity, with significant implications for cancer therapy. The integration of drug repositioning, advanced NanoBiT screening technologies, and combination therapies represents a promising strategy for precision oncology. Through these efforts, we move closer to achieving transformative treatments for cancers characterized by aberrant YAP signaling.

## Supplementary Material

Supplemental Material
