# Supplementary figures and images for: Identification of YAP regulators through high-throughput screening and NanoBiT-based validation-drug repositioning for cancer therapy
Source: Anim Cells Syst (Seoul). 2025 May 8;29(1):325–38. doi: 10.1080/19768354.2025.2489389 (PMC12064127; doi:10.1080/19768354.2025.2489389)

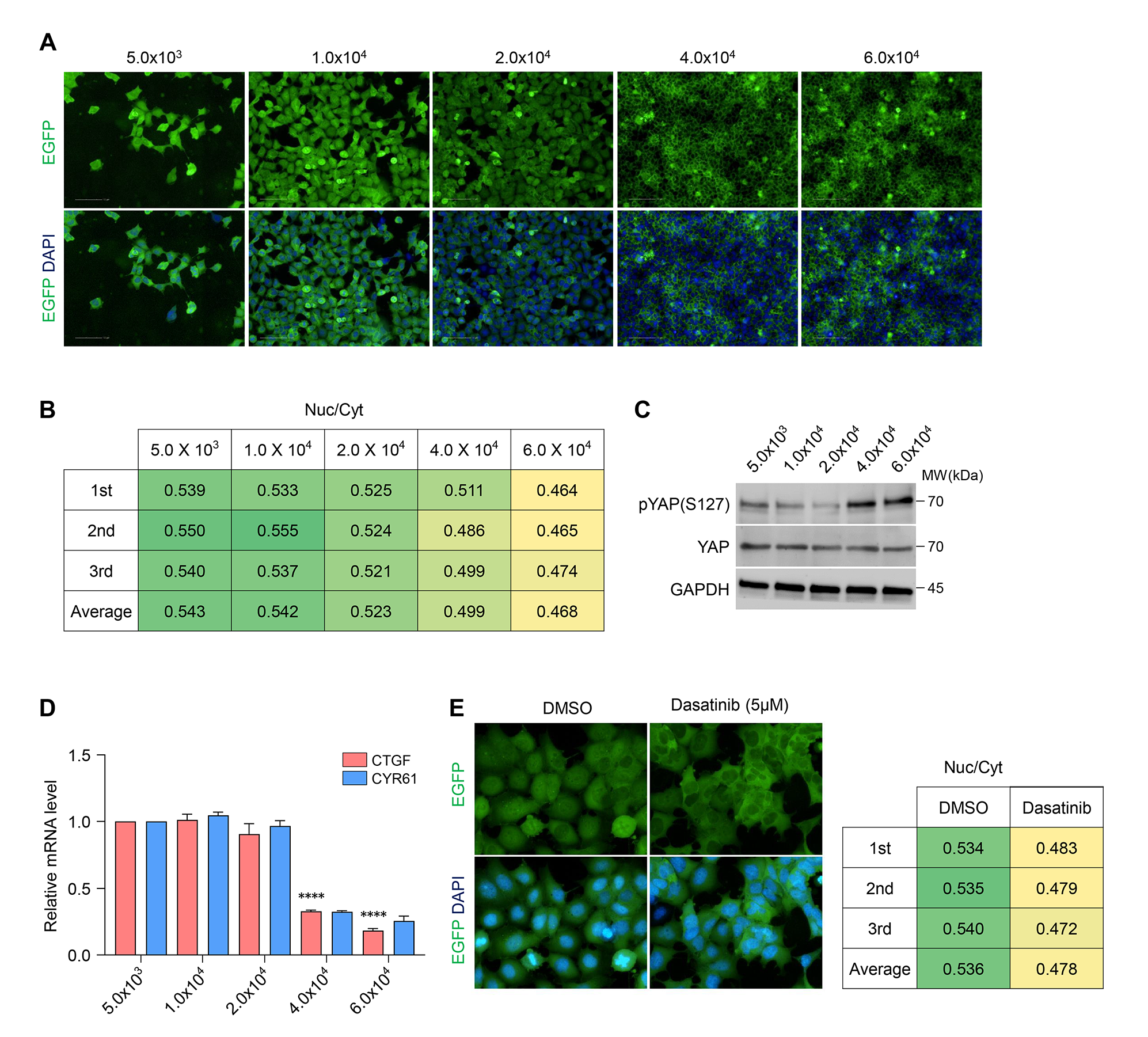

Supplement: Supplemental Material [file TACS_A_2489389_SM0582.zip › Supplementary Figure 1.tif]

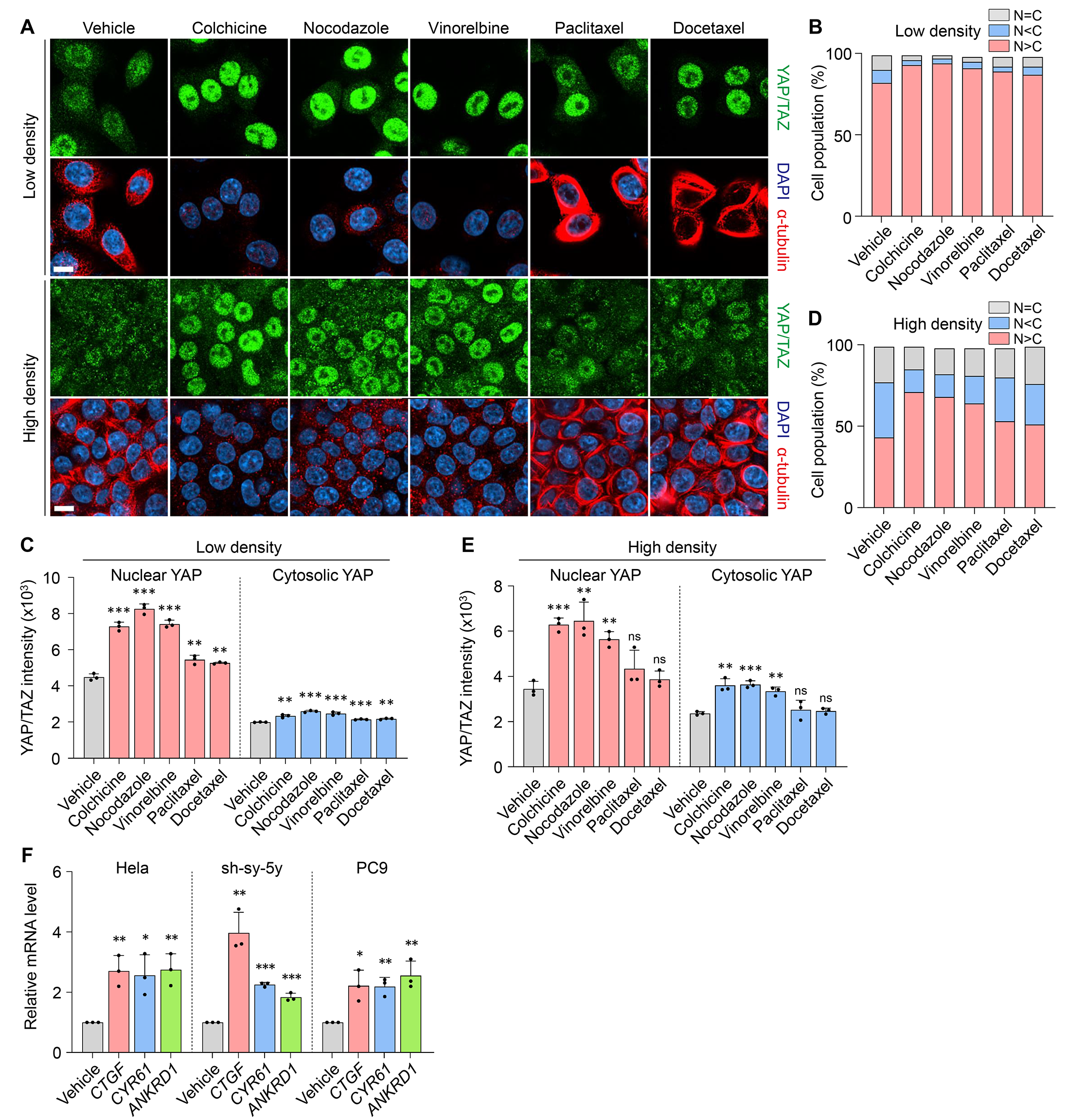

Supplement: Supplemental Material [file TACS_A_2489389_SM0582.zip › Supplementary Figure 2.tif]
